# Model Systems in Endometriosis Research: Translation, Translation, Translation!

**DOI:** 10.3389/frph.2021.809366

**Published:** 2022-01-11

**Authors:** Patrick Groothuis

**Affiliations:** Department of In Vivo Pharmacology, Toxicology and DMPK, Byondis B.V., Nijmegen, Netherlands

**Keywords:** translational research, primary tissue, organoids, PDX models, PKPD

## Introduction

Endometriosis is a disease characterized by the presence of tissue resembling endometrium (the lining of the uterus) outside the uterus, and is associated with aberrant menstrual bleeding, cyclic and chronic pelvic pain, and a reduced capacity of the women to conceive (1–3). Endometriosis has a severe impact on women's quality of life, work productivity, sexual relationship, and self-esteem (4–7), and the cost associated with the treatment of endometriosis in referral centers, equals that of other chronic high-impact disorders such as diabetes mellitus, Crohn's disease, rheumatoid arthritis, and migraine (8, 9). Despite this, endometriosis is hardly recognized as high-impact disorders by general practitioners, society, funding organizations, and pharmaceutical industry.

The list of therapeutics in development for endometriosis does seem quite impressive, but upon a more diligent look, it becomes clear that the variety of mechanisms that are targeted is quite limited and the majority of drugs still aim to suppress the hypothalamic–pituitary–gonadal (HPG) axis and steroid hormone activity, whereas the few drugs aimed at less traditional targets, such as for instance interleukin 1 receptor associated kinase 4, prolactin receptor, sirtuin-1, Jun kinase, and the P2X purinoceptor 3, have yet to demonstrate their promise. It remains elusive why, despite the numerous technological advancements in the past decades, we do not see more noteworthy innovative therapeutics in the pharmaceutical pipelines with the potential to revolutionize the medical treatment of endometriosis. It does, however, attest to the fact that our understanding of the pathogenesis and, more importantly, the resilience of endometriosis, is far from complete.

Developing drugs is a risky endeavor. Companies have to deal with high attrition rates, fierce competition from rival companies, increasing demands regarding safety and efficacy, and growing costs (10). Looking at the R&D efficiency, which is defined as the total investment in R&D per novel drug that is marketed (11), it is evident that drug attrition has the most costly impact on the total R&D spending. More than 95% of programs that enter the clinic in women's health, fail in the clinical stages (12). As expected, many projects fail because of safety concerns, strategic decisions, or commercial reasons, but the majority of programs in the clinical stage actually fail because studies do not meet their efficacy endpoints (13, 14). Even in the final stages of the drug development process, about half of the programs stumble and fail to achieve approval by regulators. This is worrisome, as these drugs were carefully selected after exhaustive preclinical characterization. In other words, the model systems used apparently are not able to adequately predict human safety and efficacy.

## Why We Should Shift the Focus to Using Primary Tissues

The choice of (animal) model for basic and translational research projects is often motivated by cost, ease of access, and use, the mechanism of action under investigation, as well as by the general consensus in the research community. Many different model systems of varying complexity have been developed in the past decades and have proven valuable to elucidate mechanisms and pathways underlying the pathogenesis of endometriosis and endometriosis-associated symptoms (15). However, even though many medical treatments that are effective in women with endometriosis (mostly modulators of the production and activity of estrogen) are also active in preclinical models, there is no evidence yet that the models can also predict a positive clinical outcome of compounds that act outside the scope of the HPG axis (16). Evidently, the cells and/or tissues maintained *in vitro, ex vivo*, or *in vivo*, fail to adequately reproduce the diseased state and more attention should be focused on advancing model systems to the point that they mimic the human condition and have predictive value.

Increasing the predictive value of preclinical models is a major hurdle for all endometriosis scientists, partly because endometriosis is a complex multifactorial and heterogeneous disease that is not easy to replicate *ex vivo* or *in vivo*, but also because incorporating and monitoring clinical endpoints such as pain and fertility in translational models is cumbersome and not common practice (17). With regard to evaluating pain in animal models and the evaluation of therapeutics basically two approaches are employed. Either pain models are used in which pain is evoked and the focus is on treating the pain directly, or alternatively, disease models with endometriosis-like lesions are generated to evaluate disease-modifying treatments and pain, pain sensitization or well-being are monitored next to for instance the effects on lesion size or other pharmacodynamic parameters. Even though there is not a clear relation between the size of endometriotic lesions and the severity of the pain symptoms (18), surgical removal of the complete lesion does provide significant pain relief to the majority of women (19, 20), as do medical treatments that significantly reduce lesion burden (21, 22). This implies that effective treatments that significantly reduce lesion burden can be as effective as surgical intervention, and that it is worthwhile to invest in recreating the “disease phenotype” as accurately as possible. In view of the fact that the ectopic endometriosis tissue is fundamentally different from the endometrium and that it is important that all critical determinants of the disease are reflected in the model, the rational approach would be to use primary cells and/or tissues derived from endometriotic lesions.

## New Options to Reconstruct Human Endometriotic Lesions are Emerging

Endometriosis tissue has been utilized in various ways, i.e., for the isolation and culture of epithelial and stromal cells, as well as the preparation of tissue explants which are subsequently cultured *ex vivo* or transplanted into immunodeficient mice [reviewed by (15)]. Obvious limitations of these methods are that in the cell cultures the interaction with the other components that are part of the local “disease environment” is lacking, that epithelial cells and tissue explants can only be cultured short term and cannot be passaged, and that the lesions are generally extremely heterogeneous consisting of not only glands and stroma but also large areas of fibromuscular/fibrotic tissue. Some groups have succeeded in immortalizing endometriotic cells (23, 24) to prolong the lifespan of the cells, but despite the fact that certain mechanisms characteristic to endometriotic lesions are maintained, and the fact that they can form 3-dimensional heterotypic spheroids when combined with immortalized endometrial stromal cells (25), there is no data yet to support that they have any translational value. The same can be said for the *ex vivo* tissue cultures and patient-derived mouse xenograft models, even though fundamentally, the explants do contain all components of the endometriotic lesion, including the extracellular matrix, resident immune cells, and blood vessels.

In cancer research the use of patient-derived xenograft (PDX) models are already widely accepted, and co-clinical trials in which tumor tissue collected at surgery is grafted in mice (also referred to as “avatar mice”) and the patient and the mice were treated with the same regimen, show a very good concordance in response (26–28). This is in line with the fact that short term culture of tissue in mice has little impact on the genotype and phenotype of the tissue (27). The grafting success rates of primary tissue explants prepared from different lesion types are quite good, however, the immunodeficient background of the recipient mice and the fact that within 2 weeks the human stroma and blood vessels are replaced by murine cells (29, 30), limit the period of time the (epi)genetic and phenotypic make-up of the human endometriotic tissue may be maintained. Moreover, the logistic challenges of tissue collection and typing, as well as lesion heterogeneity may yet prevent PDX models of becoming the gold standard translational model, unless off course the models prove to be predictive after all.

In the meantime the diligent and persistent search for new ways to recreate the “disease environment” has entered a new era now that investigators finally have succeeded to generate organoids not only from cancerous tissues (31), but also from reproductive tissues, including normal endometrium (32, 33) and endometriosis tissues (34, 35). This opens a whole new avenue of possibilities because of the unique properties of these structures. Organoids have the (epi)genetic make-up (35) mimic the physiological responses of the tissue, and can be stored, expanded, and propagated for extended periods of times, thus offering the possibility of creating a biobanking resource from clinical biopsies (33, 34). Despite this exciting breakthrough, there are still quite some challenges ahead, the most important one being to complement the organoids with other components of the microenvironment in order to better mimic real life conditions (36).

The components of endometriotic (and adenomyotic) lesions have been quite well-described, and consist of endometrium-like tissue consisting of glands and stroma, recently also referred to as lesion-initiating cells (36), surrounded by the lesion microenvironment comprising fibromuscular or myometrial muscle cells, nerves, blood vessels, immune cells, and extracellular matrix. However, besides combining all cellular components in a 3-dimensional model, Gnecco et al. also stressed the importance to model the disease-defining dynamic pathophysiological processes (36). Examples of such dynamic behaviors in the lesion are for example proliferation and invasion of lesion-initiating cells, smooth muscle hyperplasia, influx and activation of immune cells, recruitment of vasculature, enhancement of sensory innervation, and stiffening of the local microenvironment, as well as responsiveness to external cues such as steroid hormones, nutrients, and inflammatory cytokines.

The most apt platforms to engineer the 3-dimensional microenvironment and model the most relevant aspects of endometriosis in a reproducible manner, are micro- and meso-fluidic “organ on chip” technologies, well-known for their use in for instance toxicological studies and building perfused microvascular networks (37–39). Co-culturing endometriotic organoids with lesion-derived stromal cells, as well as immune and fibromuscular cells, in synthetic extracellular matrices on these microfluidic platforms may enable the reconstitution of a microvascularized and innervated lesion-like environment (40, 41).

## Don't Forget to Pay Attention to the Exposure-Response Relationship

The *in vitro* patient-derived models are expected to be very useful for the identification and validation of new targets, confirm target binding/modulation, screen novel therapeutics, and possibly identify key (surrogate) biomarkers for use in clinical trials. However, in order to translate these observations to a clinical application, *in vivo* pharmacology studies are indispensable (42, 43). It is important to demonstrate that the drug gets to the site of action upon dosing in the same fashion as patients will be dosed later, and to describe the *in vivo* relationship between the exposure (pharmacokinetics or PK) and the induction of the desired pharmacological effect of the lead compounds and their toxicities (pharmacodynamics or PD) in a PKPD model, in order to assess whether a sufficient therapeutic window can be achieved in humans. An added benefit of the organoids in this regard is that they are well-suited for *in vivo* transplantation (34), and in contrast to PDX models, they can be generated with great uniformity and reproducibly from biobanked samples generated from different lesion types and stages, which potentially will dramatically increase the robustness of and the accessibility to the model systems.

## Conclusion

Combining the exciting innovations in tissue engineering and 3-D microfluidics systems with components derived from primary endometriotic tissues may allow investigators to mimic the disease environment and holds great promise for the future of translational research in endometriosis. Such models will allow the evaluation and selection of therapeutic modalities with the potential to modify or eradicate endometriotic cells or lesions. However, whether the new generation model systems can also predict clinical efficacy of novel drugs in the patients. Meanwhile, scientist should increase their efforts to incorporate PKPD modeling into their work flows.

## Author Contributions

The author confirms being the sole contributor of this work and has approved it for publication.

## Author Disclaimer

The views and opinions expressed in this manuscript are that of the presenter and do not represent the thoughts, intentions, plans, or strategies of Byondis B.V.

## Conflict of Interest

PG is an employee of Byondis B.V.

## Publisher's Note

All claims expressed in this article are solely those of the authors and do not necessarily represent those of their affiliated organizations, or those of the publisher, the editors and the reviewers. Any product that may be evaluated in this article, or claim that may be made by its manufacturer, is not guaranteed or endorsed by the publisher.
